# Anemia, a moderate public health concern among adolescents in South Ethiopia

**DOI:** 10.1371/journal.pone.0191467

**Published:** 2018-07-17

**Authors:** Mohammed Feyisso Shaka, Yohannes Addisu Wondimagegne

**Affiliations:** Department of Public Health, College of Health Sciences and Medicine, Dilla University, Dilla, Ethiopia; INIA, SPAIN

## Abstract

**Background:**

Adolescence is characterized by rapid growth and development and iron requirements increase during this time. Adolescents with poor diet in early childhood and/or females with an early onset of menarche may be at greater risk for developing anemia due to the rapid depletion of iron stores in their bodies.

**Objective:**

The objective of this study was to assess the prevalence and severity of anemia among school adolescents in Wonago district, Gedeo Zone, South Ethiopia.

**Methods:**

A school based cross-sectional study was conducted among 443 randomly selected school adolescents across 15 schools (2 secondary schools and 13 primary schools) in the district. Hemoglobin concentration from a capillary blood sample was determined by portable hemoglobin meter (HemoCue). Descriptive statistics were computed for prevalence of anemia, anthropometric measurements, socio-economic and socio-demographic variables. A hierarchical multivariable logistic regression analysis was done to identify determinants of anemia among adolescents.

**Results:**

The prevalence of anemia among adolescents in the study area was 22%. Anemia was higher among those in early adolescence period (10–13 years) (AOR: 4.75, CI: 1.69–13.35) compared to late adolescence (17–19 years) and among those with height for age z-score less than -2 (AOR: 6.23, CI: 1.98–19.62). Similarly, anemia was higher among those in households with a family size greater than five members (AOR: 9.82, CI: 2.42–39.88), adolescents from rural areas (AOR: 4.37, CI: 1.54–12.46) and families who purchase food needed for daily consumption (AOR: 3.25, CI: 1.42–7.45). On the other hand, adolescents from middle wealth quintiles (AOR: 0.26 (CI: 0.07–0.98)) and female adolescents (AOR: 0.34 (CI: 0.15–0.79) were less likely to be anemic in this study.

**Conclusions:**

The prevalence of anemia in this population is of moderate public health concern, adolescents of both sexes are among groups at risk for the development of anemia. The need for further assessment of the etiology of anemia should be considered to design pragmatic intervention programs.

## Background

The World Health Organization (WHO) defines adolescents as the population in the age range of 10–19 years[1]. It is a transition period between childhood and adulthood [2] characterized by rapid growth and development [3]that offers a second and last chance for the catch up growth in life cycle[4]. Adolescents gain 30% of their adult weight and more than 20% of their adult height between 10–19 years [5]. Due to such remarkable physical growth and development, the needs for both macro and micronutrient among adolescents are considerably high[6]. This makes adolescents an important physiological group whose nutritional needs demand special attention[7].

Anemia is a condition in which the number of red blood cells (RBCs) is decreased resulting in insufficient oxygen-carrying capacity of the RBCs to meet the physiological needs of the body [8]. Three causes of anemia exist: blood loss, increased destruction of RBCs (hemolysis) and decreased production of RBCs[9]. Globally, iron deficiency is thought to be the most common cause of anemia. However, other nutritional deficiencies (including folate, vitamin B12 and vitamin A deficiencies), acute and chronic inflammation, parasitic infections, and inherited or acquired disorders that affect hemoglobin synthesis, red blood cell production or red blood cell survival can all cause anemia[8–10].

Anemia adversely affects the cognitive performance, behavioral characteristics and physical growth of infants, preschool and school-age children. It also affects the immune status and morbidity from infections of all age groups and the use of energy sources by muscles. Hence, the physical capacity and work performance of adolescents and adults of all age groups are significantly affected[11]. Among adolescents, anemia affects not only the present health status but can also have deleterious effects in later life. The rates of low birth weight, pre-maturity, neonatal and infant mortality among children born to undernourished adolescent girls is high. Later on, these undernourished girls become anemic and produce low birth-weight babies[12].

During adolescence the need for iron increases from preadolescent level of 0.7–0.9 mg Fe/day to up to 2.2 mg Fe/day both among adolescent boys and girls. This increased iron requirement is attributable to peak pubertal development characterized by expansion of total blood volume, increase in lean body mass and the onset of menstruation in adolescent females[4]**.** Iron need in females continue to remain high after menarche due to menstrual blood loss where the iron need averages about 20 mg of iron per month and it may also be as high as 58 mg in some individuals[13].

In developing countries parasitic infections and other infectious diseases are prevalent which increases the requirement of iron in human body[14]. Poor quality of diet consumed during early childhood[15] and early onset of menarche[16], can lead to depletion of iron stores**. A s**ignificant number of adolescents, specifically females, have an iron intake of only 10–11 mg/day which accounts for about 1 mg of iron absorption into the body[4]. This condition is further complicated when adolescents are exposed to frequent infections, heavy workload or increased physical activity and early pregnancy in females [17].

Multiple and multifaceted factors contribute to anemia, with iron deficiency being the most common cause. Evidence from the literature suggests that female adolescents[18,19]; adolescents in early stage of adolescence period, females in a physiological state like menstruation, adolescents from low socio-economic status, adolescents with macronutrient deficiency[18,20–22] and adolescents with malaria and helminth infection[20,23,24]are more prone to the development of anemia. Additionally, the area of residence (rural or urban) and meal patterns [22,25], family food sources[26] family size[22] and educational status of families[19] are common factors associated with anemia particularly in developing countries.

In Ethiopia, while sexually transmitted diseases and other reproductive health issues of adolescents have been given considerable importance, limited work has been done on adolescents’ nutritional status. Information regarding adolescents’ nutritional status and burden of anemia among all adolescents and particularly among adolescent boys is not readily available. Nutrition-related efforts in the country put much emphasis on early childhood and pregnancy and lactation periods while adolescence is also found to be a critical age where anemia is a major public health concern. This study aimed to assess the prevalence of anemia among adolescents to develop evidence that can assist with developing of pragmatic intervention program.

## Methods

### Study area and period

A school based descriptive cross-sectional study was conducted from March 22 –April 28, 2016 among school adolescents **(10–19** years**)** from 15 randomly selected schools in Wonago district of Gedeo zone, South Ethiopia. Wonago is one of the most densely populated area in the country with up to 1000 people per square kilometer in some parts of the district[27]. There are 23 schools in the district out of which 15 schools (2 secondary schools and 13 primary schools) were randomly selected for this study.

### Sample size determination and sampling technique

The sample size was determined using the a formula for single population proportion based on the following assumptions: estimated 50% prevalence (p) of anaemia (due to absence of study on prevalence of anemia among adolescents in comparable area), a 95% confidence interval for the true prevalence and a relative precision (d) of 5%.

The formula adopted was **Z**_**α/2**_^**2**^ **p (1-p)/d**^**2**^ where Z_α/2_^2^ = 1.96, p = 0.5 and d = 0.05. The calculated sample size (based on this formula) was 385. Assuming a non-response rate of 15% (58 students) the final sample size for the study became 443. The schools were selected using simple random sampling method through table of randomly generated numbers. Proportional allocation method was used for determining of the number of adolescent students included from each school. The selection of each study subject (student) at each school was conducted by table of random numbers based on the students list available in the schools registry (roster) after excluding non-adolescent students. Terminally ill adolescents, adolescents with known chronic illness and pregnant adolescents were excluded from the study.

### Data collection procedure and tool

The questionnaire was adapted from the WHO nutritional survey[15] and peer reviewed published literature. After pretest was conducted, further modification was also made to familiarize the tool with the study setting. The questionnaire mainly contains closed-ended questions and it has also a few open ended questions used to assess the adolescents’ awareness about anemia. The tool was previously used in different studies conducted among school age children and adolescents[28–31]. Fourteen diploma laboratory technologists were employed as data collectors and three BSc laboratory technologists were assigned as supervisors. They were selected from the catchment area based on their previous experience on research. A brief training on the objective of the study, interview techniques and techniques of measurement was given for the data collectors and supervisors. Information about the study was also given for adolescent students at each school. After selection of eligible adolescents at each school some of the selected adolescents were requested to come with their parents the next day and the others were given a consent form to take home for their parents to sign on the form as evidence that the parents allowed the adolescents to participate in the study. After consent was obtained from parents the selected adolescents were interviewed by the data collectors.

### Measurement

#### Blood test

A portable hemoglobin meter (HemoCue AB, Angelhom, Sweden) was used to determine hemoglobin concentration from a capillary blood sample collected aseptically by sterile single-use disposable lancet from the fingertip. The necessary safety measures were taken during blood collection. Anemia status of the adolescent was classified based on WHO classification using hemoglobin level of the respondents. For all adolescents age ≤11 years hemoglobin value <11.5g/dL, for all adolescents age 12–14 and female adolescents ≥15 years of age hemoglobin value of less than 12 g/dL and for male adolescents > 15 years of age hemoglobin value of less than 13 g/dL.

Similarly severity of anemia was classified based on WHO classification method as: severe anemia: Hgb level below 7 g/dL, moderate anemia: Hgb level 7 g/dL—9.9 gm/dL and mild anemia: Hgb level 10 g/dL -11.4 g/dL in adolescents aged ≤11 years and Hgb level 10g/dL -11.9 g/dL in all adolescents of 12–14 years of age and female ≥ 15 years and Hgb level 10g/dL -12.9 g/dL in male ≥15 years [8,32].

#### Anthropometric measurements

Measurements of height and weight were taken according to the WHO’s guideline. Weights of the school adolescents were measured to the nearest 0.1 kg on a battery powered digital scale (SECA, UNICEF, Copenhagen) and height was measured to the nearest 0.1 cm using a wooden height-measuring board with a sliding head bar following standard anthropometric techniques [33]. Anthropometric indicators used in this study are BMI for age z-score (BAZ) and height for age z-score (HAZ). School adolescents below -2 HAZ score were classified as stunted and those with BAZ score less than -2 were classified as thin.

#### Stool examination

For the assessment of helminthes infection, containers were distributed to each adolescent and they were asked to collect and bring a sample of their feces at the time of data collection. These samples were stained and examined within 1 hr of staining by Kato-Katz method for the presence of geo-helminths (hookworm, ascaris lumbricoides, trichuristrichuria and strongloides stercoralis). Standard operating procedures (SOPs) and manufacturers’ instructions were strictly followed for all laboratory activities and reagents were checked for their expiry date.

#### Wealth index

Household’s assets that are durable and semi-durable goods were used to describe household economic status. Household questionnaire that was used by Ethiopian demographic and health survey to measure household’s socioeconomic status were applied to assess the wealth index. The wealth index was calculated by principal component analysis using data of household’s assets and the respondents’ families were classified into five according to wealth status score.

### Data analysis

For data entry, template formats were prepared in Epidata version 3.1 and data was entered by single entry. The data was exported to IBM SPSS Statistics version 20 for analysis. Descriptive statistics were computed for prevalence of anemia, anthropometric measurement, socio-economic and socio-demographic variables. Binary logistic regression was used to identify variables associated with anemia among adolescents using odds ratio (OR) and 95% confidence interval.

A hierarchical multivariable analysis based on conceptual model adapted from De Silva *et al*.[34] was used to assess independent predictors of anemia among adolescents using odds ratio (OR) with their corresponding confidence interval (CI). The first dimension of the hierarchical model includes socio-economic and environmental variables which include family size, residence of the family, wealth index, shoe wearing frequency, source of family food need, daily meal frequency, presence of regular feeding program in school and malaria endemicity in the area of residence. Variables included in the second dimension of hierarchical model were educational level of mother and educational level of father. These variables were analyzed separately from socio-economic status since they are individual characteristics of the parents while other socio-economic variables are mainly households’ characteristics. The third dimension of the model contains individual characteristics of the adolescents which include age category, sex, HAZ, BAZ and stool parasite.

The variables included in each components of hierarchical model were entered simultaneously. At each stage of hierarchical model variables having P-value ≤ 0.20 in the previous multivariable analysis were remained in the model while running the next multivariable analysis with the variables of subsequent dimension of hierarchical model. Possible interaction between variables was checked before reporting of the result of the analysis. Finally, variables with p-value <0.05 in the final stage of hierarchical model was taken as the independent predictors of anemia among adolescents in this study.

### Ethics statement

Ethical clearance was obtained from Dilla University College of Health Sciences and Medicine Ethical Review Committee and letter of permission was obtained from authorities in the study area. Informed consent was also obtained from the parent of each study subjects prior to interview and the purpose of the study was explained deeply in the consent form for the respondent parents and in addition it was explained orally for the study subjects. Confidentiality of the information obtained was assured and privacy of the respondents was also maintained.

## Results

### Descriptive and bivariate analysis result

From a total of 443 students expected to participate in the study, 424 (96%) responded for the enquiry. More than half of the participants, 247 (58.3%), were male and 180 (42.4%) of the participants were in middle period of adolescence (14–16 years). In this study about 22% of the participants were anemic and almost all 422 (99.5%) of them were with mild type of anemia. Sixty two (14.6%) of adolescents have HAZ < -2 and 11.6% have BAZ <-2. Among the study participants there was no any adolescent with BAZ greater than +2. From the stool examination results, nearly 60% of the adolescents have at least one parasite and the predominant parasite was askaris which was identified in the stool of 30% of adolescents and 5.2% of the adolescents had hook warm. Regarding the association between individual characteristics and anemia among adolescents, age category and HAZ were found to be significantly associated with anemia on bivariate analysis (Table 1).

**Table 1:** Individual characteristics of the respondents and prevalence of anemia among adolescents in Wonago district, Gedeo Zone, South Ethiopia, April 2016.

Variables	variables Category	Frequency (%)	Prevalence of anemia			
			%	OR	95%CI	P-Value
**Age**	Early adolescence (10–13)	173 (40.8)	39.9	5.23	2.36–11.58	<0.001
	Middle adolescence (14–16)	180 (42.4)	8.3	0.72	0.29–1.77	
	Late adolescence (17–19)	71 (16.7)	11.3	1.00	Reference	
	Mean age	13.9±2.3				
**Sex**	Male	247 (58.3)	24.3	1.00	Reference	0.13
	Female	177(41.7)	18.1	0.69	0.43–1.11	
**HAZ**	≥-2	362 (85.4)	19.6	1.00	Reference	0.01
	<-2	62 (14.6)	33.9	2.1	1.17–3.77	
**BAZ**	≥-2	375 (88.4)	22.1	1.00	Reference	0.55
	<-2	49 (11.6)	18.4	0.79	0.37–1.70	
**Stool parasites**	Yes	248 (58.5)	18.9	1.47	0.92–2.34	0.10
	No	176 (41.5)	25.6	1.00	Reference	

BAZ: Body mass index-for age z-score

HAZ: Height-for-age z-score

Regarding family characteristics, 286 (67.5%) of the respondents’ family live in rural area and about90% of the adolescents are from households with family size of five and above. Information about the educational status of family of adolescents show that 337 (79.4%) of fathers and 219 (51.7%) of mothers have primary education and above. About 116 (27.4%) of the respondents reported that their usual daily meal frequency was two times or below. For about half of the respondents (214 adolescents), their family produces/cultivates the food needed for household daily consumption by their own. In the school of 245 (57.8%) adolescent students, there is a regular feeding program for the last six month. On bivariate analysis educational level of parents, family size, place of residence, shoe wearing frequency, daily meal frequency and family food source were significantly associated with anemia among adolescents with P-value <0.001(Table 2).

**Table 2:** Parental characteristics and socio-economic and environmental variables and their association with anemia among adolescents in Wonago district, Gedeo Zone, South Ethiopia.

Variables	variables Category	Frequency (%)	Prevalence of anemia			
			%	COR	95%CI	P-Value
**Mother education**	Illiterate	205 (48.3)	30.7	2.44	0.97–6.12	<0.001
	Primary	180 (42.5)	12.8	0.81	0.30–2.13	
	High school and above	39 (9.2)	15.4	1.00	Reference	
**Father education**	Illiterate	87 (20.5)	39.1	5.20	2.30–11.76	<0.001
	Primary education	255(60.1)	19.2	1.93	0.90–4.12	
	High school and above	82(19.3)	11.0	1.00	Reference	
**Family size**	Four and below	43 (10.1)	7.0	1.00	Reference	<0.001
	Five to seven	219 (51.7)	32.9	6.53	1.95–21.83	
	Eight and above	162 (38.2)	10.5	1.56	0.44–5.60	
**Wealth index**	Lowest	83 (20.6)	22.9	0.95	0.44–2.02	0.14
	Second	80 (19.9)	26.3	1.14	0.54–2.40	
	Middle	76 (18.9)	10.5	0.38	0.15–0.94	
	Fourth	96 (23.9)	18.8	0.74	0.34–1.57	
	Highest	67 (16.7)	23.9	1.00	Reference	
**Place of residence**	Urban	138 (32.5)	6.5	1.00	Reference	<0.001
	Rural	286 (67.5)	29.0	5.86	2.85–12.07	
**Shoe wearing frequency**	Never or rarely	115 (27.1)	42.6	5.37	2.99–9.67	<0.001
	Some times	136 (32.1)	16.2	1.40	0.73–2.66	
	Always	173 (40.8)	12.1	1.00	Reference	
**Daily meal frequency**	Two times or below	116 (27.4)	37.1	3.11	1.92–5.06	<0.001
	Three times and above	308 (72.6)	18.9	1.00	Reference	
**Sources of Family food need**	Grow their own	214 (50.5)	11.2	1.00	Reference	<0.001
	Buy/purchase	189 (44.6)	33.9	4.05	2.41–6.82	
	Subsidies/food aid	21 (5.0)	23.5	1.86	0.58–5.99	
**School feeding**	Yes	245 (57.8)	24.5	1.00	Reference	0.10
	No	179 (42.2)	17.9	0.67	0.41–1.09	
**Malaria endemicity**	Yes	241 (56.8)	21.2	1.00	Reference	0.76
	No	183 (43.2)	22.4	1.08	0.98–1.71	

### A hierarchical multivariable analysis result (Predictors of anemia among school adolescents)

The first stage of hierarchical multivariable analysis revealed that, adolescents from households with family size of five to eight [OR: 10.60, CI: 2.40–46.79], rural adolescents [OR: 6.63, CI: 2.65–16.57], those who never or rarely wear shoes [OR: 10.39, CI: 4.29–25.18], those whose their daily meal frequency was two times or less [OR: 4.50, CI: 2.24–9.04] and adolescents from families who achieve their food need through purchasing [OR: 3.13, CI: 1.50–6.55] were found to have a higher risk of anemia. In the second stage of hierarchical model, parental characteristics (educational level of mother and father) were entered into the model. At this stage both variables were found to have no effect on anemia among adolescents in this study. However, wealth index which have no any effect on the first stage was turned to have association with anemia and adolescents in lowest wealth quintile became more likely to be anemic when compared to those in highest wealth quintile [OR: 3.60, CI: 1.13–11.49].

In the third stage of the model individual characteristics of students were simultaneously entered into the model with variables having p-value less than 0.20 in previous model. Among individual characteristics entered at this stage, adolescents in early period of adolescence [OR: 4.75, CI: 1.69–13.35] and adolescents with height for age z-score <-2 [OR: 6.23, CI: 1.98–19.62] were found to have greater risk of anemia whereas female adolescents were less likely to be anemic when compared to males [OR: 0.34, CI: 0.15–0.79](Table 3).

**Table 3:** Predictors of anemia among school adolescents in Wonago district, Gedeo Zone, South Ethiopia, April 2016.

Variables	1^st^ stage		2^nd^ stage		3^rd^ stage	
	OR (95 CI)	p-value	OR (95 CI)	p-value	OR (95 CI)	p-value
Family size		0.001		<0.001		0.003
Four and below	Reference		Reference		References	0.05
Five to seven	10.60(2.40–46.79)		15.09(3.70–61.48)		**9.82(2.42–39.88)**	
Eight and above	3.87(0.788–19.01)		6.20(1.33–29.02)		4.51(0.98–20.78)	
Wealth index		0.097		0.01		0.04
Lowest	1.85(0.67–5.09)		3.60(1.13–11.49)		2.43(0.70–8.46)	
Second	1.09(0.41–2.89)		1.69(0.57–5.02)		1.19(0.36–3.93)	
Middle	0.37(0.12–1.13)		0.39(0.12–1.30)		**0.26(0.07–0.98)**	
Fourth	1.38(0.50–3.82)		1.93(0.60–6.23)		2.29(0.70–7.57)	
Highest	Reference		Reference		Reference	
Residence		<0.001		0.003		0.01
Urban	Reference		Reference		Reference	
Rural	6.63(2.65–16.57)		4.62(1.71–12.48)		**4.37(1.54–12.46)**	
Shoe wearing		<0.001		<0.001		<0.001
Never or rarely	10.39(4.29–25.18)		12.37(4.69–32.66)		**8.15(3.08–21.57)**	
Some times	1.64(0.65–4.15)		2.19(0.79–6.05)		0.54(0.18–1.65)	
Always	Reference				Reference	
Meal frequency		<0.001		<0.001		<0.001
Two times or below	4.50(2.24–9.04)		5.09(2.40–10.79)		**4.48(2.04–9.85)**	
Three times or more	Reference		Reference		Reference	
Food source		0.002		0.001		0.02
Grow their own	Reference		Reference		Reference	
Buy/purchase	3.13(1.50–6.55)		4.01(1.81–8.88)		**3.25(1.42–7.45**)	
Subsidies/food aid	0.80(0.18–3.51)		0.89(0.18–4.32)		2.92(0.54–15.75)	
School feeding		0.27		-	-	
Yes	Reference					
No	1.45(0.75–2.80)					
Malaria endemicity		0.78		-	-	
Yes	Reference					
No	0.91(0.46–1.80)					
Father education		-		0.77	-	
Illiterate			1.15(0.32–4.06)			
Primary			0.84(0.26–2.74)			
secondaryor above			Reference			
Mother education		-		0.40	-	
Illiterate			2.17(0.36–13.13)			
Primary education			0.45(0.07–2.82)			
secondary or above			Reference			
Age category (adolescence period)		-	-			<0.001
Early (10–13)					**4.75(1.69–13.35)**	
Middle (14–16)					0.50(0.15–1.69)	
Late (17–19)					Reference	
Sex		-	-			0.01
Male					Reference	
Female					**0.34(0.15–0.79)**	
HAZ		-	-			0.002
≥-2					Reference	
<-2					**6.23(1.98–19.62)**	
BAZ		-	-			0.10
≥-2					Reference	
<-2		-	-		0.32(0.08–1.23)	
Stool parasite						0.69
Yes					Reference	
No					1.16(0.55–2.47)	

## Discussion

Anemia affects a large proportion of human population and adolescents are among the disproportionately affected population segments. In this study the prevalence of anemia is 22% and almost all (99%) of the anemia was of mild type. According to the WHO classification for public health significance of anemia based on the prevalence of anemia, the magnitude in this study is of moderate public health significance. The result of this study is comparable with the 23.6% prevalence of anemia among 10–14 years age adolescents reported in the primary school of Kersa district, Eastern Ethiopia[31]. However this prevalence is higher than the finding of a study done among school adolescents in Bonga town, Southwest Ethiopia, which found 15.2% prevalence of anemia[29]. Similarly it is higher than the 13.6% prevalence among 12–15 years adolescents in Filtu Town, Somali region, Southeast Ethiopia[20]. This may be due to differences between study participants, where the majority of the participants in our study were from rural areas and those in studies done in Bonga town and Filtu town were from urban areas. The prevalence of anemia among adolescents in this study is also nearly twice the national prevalence among youths which is 13.7%[35]. Likewise, the prevalence in this study is much higher than the 5.2% prevalence from a study conducted in Denizli, Turkey [36].

When compared to the findings on prevalence of anemia among adolescents in other countries of the world including India, Nepal, Bangladesh and Pakistan, the prevalence of anemia among adolescents in Ethiopia is relatively low. According to some studies in India the prevalence of anemia among adolescents ranges from 30–56% [37,38] in an study conducted by Hyder *et al*[39] in rural Bangladesh found anemia prevalence among adolescents to be 69%. Another study conducted by Al-Buhairan[40] found 42% prevalence of anemia among adolescents in Pakistan and Adhikari and Regmi also found a similar figure (42%) among rural Nepalese adolescents of both sexes[41]. In another study conducted in Morang district, Nepal, the prevalence of anemia among adolescent males and females was 47.7% and 52.3% respectively [42]. This lower level of anemia among adolescents in the current study compared to findings from other developing countries may be due to high iron content [43–45] of the main local food source in the area -enset (*Enseteventricosum)* [46] and the higher meat consumption behavior among communities in the study area.

From the result of the third stage (last stage) of hierarchical multivariable analysis, adolescents in early period of adolescence (10–13 years) were about five times more likely to be anemic compared to older adolescents (17–19). This result is relatively in agreement with the finding of a study from Iraq[19] where the prevalence of anemia is found to be higher among younger adolescents compared to older ones. The study also revealed that female adolescents were less anemic when compared to male adolescents, the likelihood of being anemic decreased by 66% for female adolescents. The result of this study is in agreement with findings from a study conducted in Palestine, West bank[47]. However the finding was on the contrary to studies done elsewhere[35,36,38,42,48] showing that female adolescents are more anemic as compared to males due to their higher physiologic need of iron during menstruation. Nevertheless, menstruation was not significantly associated with anemia status in this study. The lower risk of having anemia among females in this study may be attributed to more time spent at home when compared to male adolescents. In rural areas of Ethiopia female adolescents spend most of their out of school time at home and consequently they may have better chance to have more meals per day. Adolescent males are more likely to be out of home with their peers missing the meal time. However, other study in Southwest Ethiopia found no sex difference for the prevalence of anemia among adolescents[49].

Regarding the association between nutritional status and anemia among adolescents, those with HAZ <-2 were about six times more likely to be anemic. This finding is also in agreement with studies conducted elsewhere including studies conducted in Ethiopia[20,29]. Family size is also one of the socio-economic variables determining the prevalence of anemia in this study. As a result, adolescents from households with family size of five to eight are nearly 10 times more likely to be anemic when compared to those from lower family size. The result of this study is in accordance with other studies done in different parts of the world[19,29]. In a study conducted among school adolescents in Bonga town in South Ethiopia, children from households with family size above five were about three times more likely to be anemic[29]. But a study done among school children in Southwestern Ethiopia, Jimma town[50] and other study among primary School children in Eastern Ethiopia, Kersa district [31] found no significant association between anemia and family size. On the other hand, adolescents from households with family size above eight members show no significant difference with those from lower family size. This may be due to the fact that households with larger family size have more independent members that could support the family income to improve the adolescents feeding.

Other socio-economic factors determining the prevalence of anemia among adolescents in this study were area of residence and wealth index. Adolescents from rural areas were more than four times at higher risk of anemia compared to urban adolescents. This may also be the reason why the prevalence of anemia in this study is higher than findings from other studies conducted in urban areas of Ethiopia. Regarding wealth index adolescents from families in the middle wealth quintile were less likely to be anemic when compared to those from highest wealth quintile. The likelihood of having anemia decreased by 74% for adolescents from families with middle wealth quintile. This is on the contrary to other studies conducted in different parts of the world [29,36,50] where usually lower economic status increases the risk of anemia due to higher possibility of food shortage. The unusual lower prevalence of anemia among adolescents from middle wealth quintile in this study may be due to the fact that, the area is a cash crop area and most of the time households with higher socio-economic status in this area are merchants. The source of family food need for such households is purchasing from the market and adolescents from households who achieve their food need through purchasing were more likely to be anemic in this study.

Shoe wearing frequency was also associated with the risk of anemia among adolescents in this study. Adolescents who do not wear shoes at all or wear them rarely were eight times more likely to be anemic, those who do not wear shoes may be from low socioeconomic status which can be associated with malnutrition. Parasite infestation, particularly geohelminth infestation with hookworm, ascaris lumbricoides, trichuris trichuria, E.vermicularis and strongloides stercoralis, did not show a significant association with anemia among adolescents in the current study. Likewise, a study done among adolescent school girls in Western Kenya found no significant association between anemia and parasite infestation[51].

Prevalence of anemia was also associated with daily meal frequency and source of family food need. Adolescents who eat two or less times per day were nearly five times more likely to develop anemia as compared to those who eat three or more times. This result also complies with the study done among adolescents in Multan Nagar urban slum in India[38]. This might be so because adolescent who eat three or more times per day gets more energy and have high likelihood of consuming iron reach foods. Regarding the source of family food need, adolescents from families who meet food need through purchasing were about three times more likely to be anemic when compared to those who grow by their own. This might be due to the fact that those who grow by their own produce more food with variety and amount and their children could get well balanced and sufficient diet.

This study explored the status of anemia in a segment of the population understudied, specifically male adolescents with adequately representative sample and probable method of sampling procedure. However, we acknowledge certain important limitations, one of the main limitations of this study was that we did not explore different etiologies of anemia which is important for designing specific and appropriate intervention mechanisms for each etiologic factor. Another limitation was the absence of data on variables like dietary diversity that may show how variation in the consumption of variety of food sources determines the prevalence of anemia in the study area. Another limitation of this study was the exclusion of adolescents who are out of school which could have different nutritional status when compared to in school adolescents. This may affect the generalizability of the result of the study for all adolescents. However, since the number of adolescents who are out of school is relatively very small when compared to school adolescents, this study could also give a useful insight into the prevalence of anemia in this population.

## Conclusion and recommendation

The prevalence of anemia in this population is of moderate public health concern and the result implies that adolescents of both sexes are among groups at risk for the development of anemia. Further studies to identify the typical etiologies of anemia in this population are needed to design appropriate intervention programs. Nutrition programs aiming to alleviate anemia in this population should give special focus to adolescents in early adolescence period, male adolescents and adolescents from low socioeconomic status. Increasing access to school meals and improving the quality of meals should also get due emphasis to increase the daily meal frequency of the adolescents.

## Supporting information

S1 Dataset Data underlying this study. (SAV)

## Abbreviations

AOR: **Adjusted Odds Ratio**
BAZ: Z–Score for BMI for age
BMI: Body Mass Index
COR: Crude Odds Ratio
CSA: Central Statistical Agency
DALYs: Disability Adjusted Life Years
g/dl: gram per deciliter
HAZ: Z score for height for age
IDA: Iron Deficiency Anemia
UNICEF: United Nations Children Fund
WHO: World Health Organization

## References

[1] WHO. Young People’s Health A challenge for Society. Geneva, Switzerland; 1986.

[2] WHO/UNFPA/UNICEF. The Reproductive Health of adolescents: A strategy for action- A joint WHO/UNFPA/UNICEF statement, Geneva, 1989.

[3] Giuseppina D. Nutrition in adolescence. Pediatr Rev. 2000;21:32–3. 10617761 10.1542/pir.21-1-32

[4] Beard J. Iron requirements in adolescent. J Nutr. 2000;130:4405–25. 10.1093/jn/130.2.440S 10721923

[5] Lal S. Textbook of Community Medicine (Preventive and Social medicine). 1st ed New Delhi: CBS Publishers and Distributors; 2007.

[6] S. Pee, D. Soekarjo, J. Kusin, W. Schreurs, W. Schultink, Muhilal and M. Bloem. Effectiveness of weekly vitamin A (1000 IU) and iron (60 mg) supplementation for adolescent boys and girls through schools in rural and urban East Java, Indonesia. Eur J Community Med. 10.1038/sj.ejcn.1601914 15164114

[7] Visweswara R. Vital Statistics and nutritional status Of Indians. Indian J Nutr diet. 1987;24:272–97.

[8] WHO. Haemoglobin concentrations for the diagnosis of anaemia and assessment of severity. Vitamin and Mineral Nutrition Information System. Geneva, World Health Organization, 2011 (WHO/NMH/NHD/MNM/11.1) (http://www.who.int/vmnis/indicators/haemoglobin.pdf.

[9] Maakaron JE, Taher AT, Conrad ME. Anemia Practice Essentials, Pathophysiology, Etiology. MesScape; 2016.

[10] Fauci AS. Harrison’s Principles of Internal Medicine. 18th ed McGraw-Hill Companies; 2012.

[11] Anuradha G., Rakesh K., Salhotra V. S., Mohan A. and Sheetal R. Guidelines for Control of Iron Deficiency anaemia. National Iron+ initiative, 2013.

[12] Deshpande N., Karva D., Agarkhedkar S. and Deshpande S. Prevalence of anemia in adolescent girls and its co-relation with demographic factors. Int J Med public Heal. 2013;3:235–9.

[13] Beard J. Iron biology in immune function, muscle metabolism and neuronal functioning. J Nutr. 2001;131(2S–2):568S–579S. 10.1093/jn/131.2.568S 11160590

[14] Brabin B., Hakimi M. and Pelletier D. An Analysis of anemia and pregnancyrelated maternal mortality. J Nutr. 2001;131:604–15. 10.1093/jn/131.2.604S 11160593

[15] WHO. Adolescent Nutrition: A Review of the Situation in Selected South-East Asian countries, WHO, New Delhi, 2006.

[16] Gupta, Pant B., kumari R. and G. M. Screen Out Anemia among Adolescent Boys as Well! Natl J Community Med. 2013;4(1):20–5.

[17] Martos F. and Lopez M. Iron availability: An updated review. Int J Food Sci Nutr. 2004;55:597–606. 10.1080/09637480500085820 16019304

[18] Lee J, Lee JH, Ahn S, Kim JW, Chang H, Kim YJ, et al Prevalence and Risk Factors for Iron Deficiency Anemia in the Korean Population : Results of the Fifth Korea National Health and Nutrition Examination Survey. J Korean Med Sci. 2014;29:224–9. 10.3346/jkms.2014.29.2.224 24550649 PMC3924001

[19] Al-sharbatti SS, Al-ward NJ, Al-timimi DJ, Phil M. Anemia among adolescents. 2002;(12114):189–94. 12682686

[20] Gutema B, Adissu W, Asress Y, Gedefaw L. Anemia and associated factors among school-age children in Filtu Town, Somali region, Southeast Ethiopia. BMC Hematol. 2014;14:13 10.1186/2052-1839-14-13 25170422 PMC4147173

[21] Siddharam SM, Venketesh GM, Thejeshwari HL. A Study of Anemia Among Adolescent Girls in Rural Area of Hassan district, Karnataka, South India. Int J Bioogical Med Sci. 2011;2(4):922–4.

[22] Gupta D, Pant B, Kumari R, Gupta M. SCREEN OUT ANAEMIA AMONG ADOLESCENT BOYS AS WELL ! 2013;4(1):20–5.

[23] Tatala SR, Kihamia CM, Kyungu LH, Svanberg U. Risk factors for anemia in schoolchildren in Tanga Region, Tanzania. Tanzanian J Heal Res. 2008;10(4):189–201. 10.4314/thrb.v10i4.45074 19402580

[24] Osazuwa F, Ayo OM, Imade P. Contribution of malnutrition and malaria to anemia in children in rural communities of Edo state, Nigeria. North Am J Med Sci. 2010;2(1):532–6. 10.4297/najms.2010.2532 PMC3338216 22558561

[25] Barugahara E, Kikafunda J, Gakenia W. PREVALENCE AND RISK FACTORS OF NUTRITIONAL ANAEMIA. 2013;13(3):7679–92.

[26] USAID; CORE Group; MCHIP; FHI360; FANTA III; SPRING. Conceptual Frameworks for Anemia: Multisectoral Anemia Partners Meeting. In Washington, DC; 2013.

[27] Wikipedia, the free encyclopedia, 29 January 2014. [Online]. [Accessed 15 December 2014].

[28] Tesfaye M, Yemane T, Adisu W, Asres Y, Gedefaw L. Anemia and iron deficiency among school adolescents : burden, severity, and determinant factors in southwest Ethiopia. Adolesc Health Med Ther. 2015;6:189–96. 10.2147/AHMT.S94865 26719736 PMC4687608

[29] Tesfaye M, Yemane T, Adisu W, Asres Y, Gedefaw L. Anemia and iron defciency among school adolescents: burden, severity, and determinant factors in southwest ethiopia, vol. 6, pp., 2015. Adolesc Heal Med Ter. 2015;6:189–96. 10.2147/AHMT.S94865 PMC4687608 26719736

[30] Mulugeta A, Hagos F, Stoecker B, Kruseman G, Linderhof V, Abraha Z. Nutritional Status of Adolescent Girls from Rural Communities of Tigray, Northern Ethiopia. 23 2009.

[31] Mesfin F, Berhane Y, Worku A. Anemia among Primary School Children in Eastern Ethiopia. PLoS One. 2015;10(4). 10.1371/journal.pone.0123615 PMC4406736 25902055

[32] WHO, UNICEF, UNU. Iron defciency anaemia: assessment, prevention, and control A guide for programme managers. Geneva, World Health Organization, 2001. WHO/ NHD/01.3.

[33] WHO. The WHO child growth standards: World Health Organization 2009. [Online]. http://www.who.int/growthref/en/webcite. [Accessed 20 November 2014].

[34] De Silva LSM, Giuglian ERJ, de Castro Aerts DRG. Prevalence and risk factors for anemia among children in Brazil. Rev Saúde Pública 2001;35:66–73. 11285520 10.1590/s0034-89102001000100010

[35] Ethiopia Central Statistical Agency. Ethiopia Demographic and Health Survey 2011; Preliminary Report. Calverton, MD: Addis Ababa Ethiopia: Central Statistics Agency; 2011".

[36] Balcı YI, Karabulut A, Gürses D, Çövüt İE. Prevalence and Risk Factors of Anemia among Adolescents in Denizli, Turkey. Iran J Pediatr. 2012;22(1):77–81. 23056863 PMC3448219

[37] National Nutrition Monitoring Bureau, Diet and Nutritional Status of Rural Population and Prevalence of Hypertension among adults in Rural Areas, NNMB Technical Report No. 24, 2006, NNMB, NIN, Hyderabad.

[38] Gupta D, Pant B, Kumar R. Screen out Anemia Among Adolescent Boys as Well. Natl J Community Med. 2013;4(1):20–5.

[39] Hyder SMZ, Haseen F, Khan M, Schaetzel T, Jalal CSB, Rahman M, et al A Multiple-Micronutrient-Fortified Beverage Affects Hemoglobin, Iron, and Vitamin A Status and Growth in Adolescent Girls in Rural Bangladesh 1, 2. J Nutr. 2007;137(May):2147–53. 17709456 10.1093/jn/137.9.2147

[40] Al-Buhairan A. and Oluboyede O. Determination of serum iron, total iron-bindingcapacity and serum ferritin in Saudi adults. Ann Saudi Med. 2001;21(1–2):100 17264605 10.5144/0256-4947.2001.100

[41] Adhikari R. and Regmi S. A study on the factors influencing nutritional status of adolescent girls in Nepal Nutrition of Adolescent Girl’s Research Program. 1994.

[42] Sinha A., Singh G. and Karna K. Prevalence of anemia amongst adolescents in Biratnagar, Morang District. Nepal IJPBA. 2012;3(5):1077–81.

[43] Nurfeta A., Tolera A., Eik L.O. et al Yield and mineral content of ten enset (Ensete ventricosum) varieties. Trop Anim Health Prod (2008) 40: 299 10.1007/s11250-007-9095-0 18557193

[44] Atlabachew M, Chandravanchi BS. Levels of major, minor and trace elements in commercially available enset (Ensete ventricosum (Welw.) Cheesman) food products (Kocho and Bulla) in Ethiopia. J. Food Compos. Anal.2008; 21:545–552.

[45] Abebe Y, Bogale A, Hambidge KM, Stoecker BJ, Bailey K, Gibson RS. Phytate, zinc, iron, and calcium content of selected raw and prepared foods consumed in rural Sidama, Southern Ethiopia, and implications for bioavailability. J. Food Compos. Anal.2007; 20:

[46] UNU-IAS & IR3S/UTIAS. Socio-ecological production landscapes and seascapes (SEPLS) in Africa. United Nations University Institute for the Advanced Study of Sustainability. 2016, Tokyo, pp. 28–36.

[47] Mikki N, Abdul-Rahim HF, Stigum H, et al Anaemia prevalence and associated sociodemographic and dietary factors among Palestinian adolescents in the West Bank. East Mediterranian Heal J. 2011;17(3):208–17. 21735961

[48] Kaur Kaur. A Comparison of Nutritional Profile and Prevalence of Anemia among Rural Girls and Boys. J Exerc Sci Physiother. 2011;7(1):11–8.

[49] Desalegn A, Mossie A, Gedefaw L. Nutritional Iron Deficiency Anemia: Magnitude and Its Predictors among School Age Children, Southwest Ethiopia: A Community Based Cross-Sectional Study. PLoS One. 2014;9(12). 10.1371/journal.pone.0114059 PMC4250059 25438147

[50] Assefa S., Mossie A. and Hamza L., Prevalence and severity of anemia among school children in Jimma Town, Southwest Ethiopia. BMC Hematol. 2014;1:1–13. 10.1186/2052-1839-14-3 PMC3896819 24433408

[51] Leenstra T, Kariuki SK, Kurtis JD, Oloo AJ, Kager PA, Kuile FO. Prevalence and severity of anemia and iron deficiency : cross-sectional studies in adolescent schoolgirls in western Kenya. 2004;681–91. 10.1038/sj.ejcn.1601865 15042138

